# In vivo dosimetry for proton therapy: A Monte Carlo study of the Gadolinium spectral response throughout the course of treatment

**DOI:** 10.1002/mp.17625

**Published:** 2025-01-21

**Authors:** Mariana Brás, Hugo Freitas, Patrícia Gonçalves, João Seco

**Affiliations:** ^1^ German Cancer Research Centre Heidelberg Germany; ^2^ Laboratório de Intrumentação e Física Experimental de Partículas Lisbon Portugal; ^3^ Department of Physics Instituto Superior Técnico University of Lisbon Lisbon Portugal; ^4^ Department of Physics and Astronomy University of Heidelberg Heidelberg Germany

**Keywords:** contrast agent, dosimetry, gadolinium, in‐vivo, proton therapy, real‐time, tracking, treatment plan

## Abstract

**Background:**

In proton radiotherapy, the steep dose deposition profile near the end of the proton's track, the Bragg peak, ensures a more conformed deposition of dose to the tumor region when compared with conventional radiotherapy while reducing the probability of normal tissue complications. However, uncertainties, as in the proton range, patient geometry, and positioning pose challenges to the precise and secure delivery of the treatment plan (TP). In vivo range determination and dose distribution are pivotal for mitigation of uncertainties, opening the possibility to reduce uncertainty margins and for adaptation of the TP.

**Purpose:**

This study aims to explore the feasibility of utilizing gadolinium (Gd), a highly used contrast agent in MRI, as a surrogate for in vivo dosimetry during the course of scanning proton therapy, tracking the delivery of a TP and the impact of uncertainties intra‐ and inter‐fraction in the course of treatment.

**Methods:**

Monte Carlo simulations (Geant4 11.1.1) were performed, where a Gd‐filled volume was placed within a water phantom and underwent treatment with a scanning proton TP delivering 4 Gy. The secondary photons emitted upon proton‐Gd interaction were recorded and assessed for various tumor displacements. The spectral response of Gd to each pencil beam irradiation is therefore used as a surrogate for dose measurements during treatment.

**Results:**

Results show that the deposited dose at the target volume can be tracked for each TP scanning point by correlating it with the recorded Gd signal. The analyzed Gd spectral line corresponded to the characteristic X‐ray $k_{\alpha }$ line at 43 keV. Displacements from the planned geometry could be distinguished by observing changes in the Gd signal induced by each pencil beam. Moreover, the total 43 keV signal recorded subsequently to the full TP delivery reflected deviations from the planned integral dose to the target.

**Conclusions:**

The study suggests that the spectral response of a Gd‐based contrast agent can be used for in vivo dosimetry, providing insights into the TP delivery. The Gd 43 keV spectral line was correlated with the dose at the tumor, its volume, and its position. Other variables that can impact the method, such as the kinetic energy of the incident protons and Gd concentration in the target were also discussed.

## 1 INTRODUCTION

Light ions, such as protons, exhibit an energy deposition profile referred to as the Bragg curve. Their deposited energy per unit of path length is higher near the end of the particles' path, at the Bragg peak.^1^ This property is highly favorable in external cancer treatment, since it ensures a reduced dose delivery at the beam entrance and a nearly zero dose at the tissue after the tumor. Thus, proton therapy has the potential to increase the conformity of dose to the tumor region and decrease the normal tissue complication probability over conventional radiotherapy.^2^, ^3^, ^4^

Despite the advantages of protons in the field of radiotherapy, proton therapy brings new challenges which limit its overall application. Between them is the need for predicting as accurately as possible the range of the primary particles, and therefore the spacial distribution of dose, during treatment planning, and the delivery process.^5^ The beam particle's range is influenced by systematic and random uncertainties. These rise from organ motion, setup and anatomical variations, dose calculation approximations, biological considerations, and treatment delivery.^5^, ^6^, ^7^ Due to the sharp dose falloff profile, deviations in the dose space distribution, caused by uncertainties, can lead to severe under‐dosing of the tumor region or/and over‐dosing of the healthy surrounding tissues. A report from AAPM Task group 105^8^ states that a 5% change in dose can result in 10‐20% changes in tumor control probability or up to 20% –30% changes in normal tissue complication probabilities if the prescribed dose falls along the steepest region of the dose‐effect curves.

Dosimetric clinical practices, such as yearly, monthly, and daily QA procedures are already mandatory in every clinical facility in order to mitigate some of the effects generating systematic uncertainties in delivered dose. To account for most of the random uncertainties, appropriate margins are added around the target volume, and robust optimization is performed when using intensity modulated proton therapy.^6^ Nevertheless, unpredictable sources of uncertainty will still be present, threatening the correct and safe delivery of the treatment plan (TP). Moreover, both margins and robust optimization increase the dose to the surrounding organs to achieve a high confidence tumor control probability.

Therefore, the ability to follow in vivo and in real time the delivery of a TP, assessing the spatial deposition of dose during each fraction, could allow to reduce the applied margins or the uncertainty width in robust optimization and contribute to a daily adaptation of the TPs.^6^ Task groups such as AAPM TG‐40^9^ and AAPM TG‐62,^10^ recognized the importance of in vivo dosimetry to identify deviations in the TP delivery and to verify the dose to critical organs, recommending access of clinics to in vivo systems.

In vivo range and dosimetric measurements are performed during treatment or shortly after it. The methods applied in clinical scenarios and which can be relevant for in vivo measurements are thermoluminescence detectors (TLD), silicon diodes, metal oxide semiconductor field effect transistor (MOSFET) or optical stimulated luminescence (OSL) detectors.^7^, ^11^ However, the use of these dosimeters is limited to the placement on the skin or within accessible body cavities. Consequently, only entrance or exit measurements can be performed, which do not provide direct information regarding the target dose. The state‐of‐the‐art methods include positron emission tomography (PET)^12^, ^13^, ^14^ and Prompt Gamma.^15^, ^16^, ^17^ However, the accuracy of the first technique is highly hindered by the short half‐lives of the most abundant positron emitters, the low activity density and washout processes, while the main challenge of prompt gamma is the detection of the secondary photons, due to their high energies, ranging from 2 to 7 MeV.

A novel approach for invivo dosimetry was recently proposed,^18^ which employs a gadolinium (Gd)‐based contrast agent as a surrogate for dose measurements. This method leverages the spectral response of Gd upon proton incidence, establishing a correlation between the observed spectral features and the delivered dose and dose rate. In this study, we propose to utilize this method for the measurement of dose during the course of a proton treatment and for the tracking of the TP delivery.

Gd is a widely used contrast agent in magnetic resonance imaging (MRI) due to the paramagnetic nature of the Gd(III) ion. When injected into the patient's body, the majority of these agents will distribute between the blood and extracellular space.^19^ Tumors are physiologically distinct from normal tissue, often exhibiting leaky vasculature, compromised endothelium, and/or an underdeveloped lymphatic drainage system. Therefore, nanosized contrast agents can passively accumulate in the tumor via its vascular permeability to macromolecules.^19^, ^20^ This results in a temporary retention of greater concentrations of the contrast agent in tumors than in healthy tissue. Therefore, Gd‐based contrast agents (GBCA) are interesting for an application where the tumor region wants to be studied against the healthy tissue.

Gd is comprised of seven naturally abundant isotopes. It presents a total neutron absorption cross section of nearly 48,800barns, mainly due to the extremely high thermal neutron cross section of the isotopes 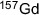 (15.7% abundance) and 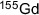 (14.8% abundance), of 254,000barns and 60,900barns, respectively.^21^ Therefore, Gd had become attractive for other applications besides its use as a contrast agent for MRI, such as Gd neutron capture therapy (GdNCT)^22^, ^23^, ^24^ or Particle Neutron Gamma‐x detection (PNGXD).^25^, ^26^ The former takes advantage of the electrons (internal conversion (IC) and Auger and Coster‐Kronig (ACK) electrons) produced upon thermal neutron capture by the 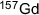 and 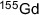 nucleus, which increase the linear energy transfer (LET) of the primary beam at this region, increasing the dose deposited locally at the tissue. In the last, PNGXD, the photon spectra produced from proton therapy due to Gd neutron capture events are used to localize the tumor, producing a dynamic tumor image to be fused with an anatomic image of the patient. Such photon spectra consist of γ‐rays of 79.5 and 181.9 keV and characteristic x‐rays of 43 keV (Kα) and 49 keV (Kβ), which also follow the 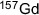 neutron capture reaction.^27^, ^28^

In this work, Monte Carlo (MC) simulations were performed to analyze the possibility of using such technique as in vivo dosimetry, observing the x‐ray spectra response to several scenarios, understanding its relationship with the irradiated volume and depth dose profile, and inspecting the possibility of tracking the delivery of the TP.

## 2 METHODS

MC simulations were carried out in Geant4 toolkit (version 11.1.1).^29^ The focus of the study was to investigate the behavior of the proposed system under different conditions and thus access its suitability for in vivo dosimetry.

### 2.1 Geometry and materials

A water cubic phantom of dimensions $10\times 10\times 10\;{\mathrm{cm}}^{3}$ was defined and placed in a vacuum environment. The volume of interest, a $2\times 2\times 2\;{\mathrm{cm}}^{3}$ Dotarem cubic box representing the tumor volume filled with a GBCA, was positioned at the centre of the phantom.

Dotarem (Guerbet) is a GBCA used in MRI of the brain, spine, and associated tissues.^30^ It consists of a 0.5 mmol/mL aqueous solution of gadoterate meglumine (empirical formula of $C_{23}H_{42}O_{13}N_{5}\mathrm{Gd}$) with a density of 1.1753 g/cm^3^ at 20 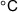.^30^

**FIGURE 1:**
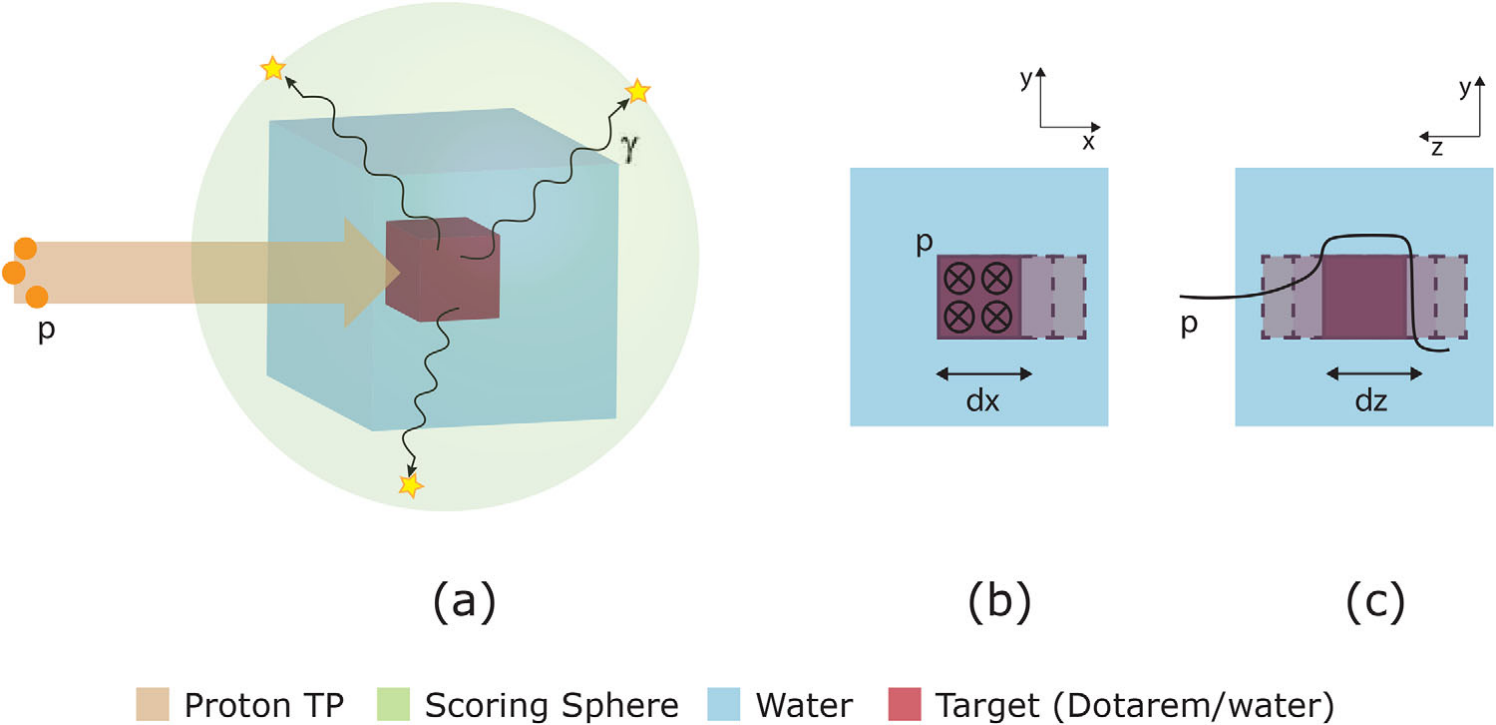
(a) Geometry implementation of the system: Cubic volume of Dotarem or water (control) representing the tumor (red) centered at a water cubic volume, representing the patient body (blue); scoring sphere surrounding the system, where the secondary x‐ and gamma‐ray photons are recorded (green); scanning proton TP as the particle source (orange). (b) Displacements of the tumor in the transversal plane, +*x* direction. (c) Displacements of the tumor in the longitudinal plane, in the upstream (+z) and downstream (‐*z*) direction. TP, treatment plan.

As control, simulations were carried with a pure water tumor cubic box, representing the tumor volume without the presence of the contrast agent. The set‐up created on Geant4 can be seen in Figure 1a.

### 2.2 Particle source

An optimized pencil beam scanning (PBS) TP was created in the open‐source toolkit matRad^31^ in order to cover uniformly the whole tumor volume with a 4 Gy physical dose. The typical multifractionated irradiation schedule is 1.8/2 Gy per fraction, although a number of studies have explored the potential benefits of alternative irradiation regimens.^32^, ^33^ For instance, in hypofractionation, higher doses are delivered per fraction over a reduced number of fractions. Doses per fraction ranging from 3 to 10 Gy were investigated, with some studies considering even higher doses.^33^, ^34^ Optimization of the TP in MatRad was performed for a proton beam, using a single fraction, with gantry angle at $0^{\circ }$ and a 3mm lateral spot spacing. A MC algorithm for dose calculation was used, the MatRad MCsquare wrapper. For the control water tumor volume, the obtained TP is composed of 539 pencil beams (PB), distributed over 11 different energy layers, ranging from 70.0 MeV to 87.81 MeV. Upon filling the target with Dotarem, the density of the volume is higher than that of water, with a value of $1.17\;g/{\mathrm{cm}}^{3}$ (when considering a pure 100% Dotarem solution in the target) as opposed to $1.0\;g/{\mathrm{cm}}^{3}$, respectively. Therefore, the TP had to be adjusted to account for the shift in proton range produced by the different densities and to ensure a comparable dose coverage within the same tumor volume. A TP composed of 588 PBs, distributed over 12 different energy layers, ranging from 70.0 MeV to 89.60 MeV were obtained. In order to achieve good statistics, while maintaining an acceptable computational running time in the Geant4 simulations, the fluence of each PB was adjusted and down‐scaled to a total of $10^{9}$ initial protons in the TP.

### 2.3 Physics

Two main mechanisms are considered to contribute to the spectral line of interest ‐ Particle Induced X‐ray emission (PIXE) and neutron capture. The physics processes and models called by the MC should respect both type of interactions. The following modules were registered to the modular physics list: (a) G4EmStandardPhysics_option4; (b) G4EmExtraPhysics, G4DecayPhysics, (c)G4RadioactiveDecayPhysics, (d) G4HadronElasticPhysicsHP, (e) G4HadronPhysicsQGSP_BIC_HP, (f) G4StoppingPhysics and (g) G4IonPhysics.

G4EmStandardPhysics_option4 is one of the most accurate combination of electromagnetic models and settings. Fluorescence from photons and electrons is activated by default, however the atomic deexcitation processes, Auger production and PIXE, were manually activated, employing the *ECPSSR_Ansto* model. This model is based on the theoretical work of Brandt and Lapicki, providing the ionization cross section of the k, L and M shells up to 5 MeV/amu, in targets with Z < 93.^35^ Out of the specified range the default Geant4 *Empirical* model is used.

G4EmExtraPhysics describes the photo‐nuclear, electro‐nuclear, synchrotron radiation, and rare electromagnetic processes. G4DecayPhysics covers the in‐flight or at rest decays of long‐lived unstable particles while G4RadioactiveDecayPhysics describes the decay of radioactive nuclei by α, $\beta^{-}$ or $\beta^{+}$ emission and electron capture. G4StoppingPhysics is employed for the possible nuclear capture at rest of negative charged particles or neutral anti‐hadrons and G4IonPhysics describes the ion processes.

The high precision (HP) models in G4HadronElasticPhysicsHP and G4HadronPhysicsQGSP_BIC_HP, treat the elastic and inelastic interactions of p, d, t, 3He and α of energy lower than 200 MeV and consider the low‐energy neutrons of energy lower than 20MeV.

### 2.4 Scoring

The whole water‐Dotarem volume was surrounded by a spherical shell of inner radius 8.7cm (scoring sphere) centered at its origin. Secondary photons originated due to proton irradiation of the volume, were scored when hitting the scoring sphere. Their energy, physics creator process, volume of creation as well as other identifying properties of the particles were recorded.

The total dose delivered to the tumor volume, by each individual PB irradiation was scored. The dose deposited by the primary protons of each PB was scored over the whole geometry in each $2\times 2\times 2\;{\mathrm{mm}}^{3}$ voxel of a mesh in a parallel plane. From this data, dose‐depth profiles were inferred as well as dose volume histograms in the tumor volume. Uncertainties in total dose and voxel dose were calculated using the history to history method.^36^

### 2.5 Researched scenarios

The Dotarem volume was displaced in incremental steps in both the transverse and longitudinal planes, in order to recreate the target movements which occur both within and between fractions. Given the simplicity and symmetry of the adopted geometry, movements in the transverse plane were represented by incremental steps of 2 mm only in the positive *x*‐axis, until a full displacement of 10 mm (Figure 1b). In the longitudinal axis, the target was displaced in 2 mm incremental steps both in the upstream (in the direction of the beam nozzle) and downstream (away from the beam nozzle) directions until a total 10 or −10 mm, respectively, displacement from the original position (Figure 1c). The previously mentioned data was recorded for each scenario. A correlation between the Gd x‐ray signal and the deposited dose at the volume was investigated.

## 3 RESULTS

### 3.1 Introducing a GBCA in the tumor volume

The irradiation of the water equivalent tumor volume with the developed TP resulted in a total dose at the target of 4.03±0.02Gy, with a $D_{90\%}$ of 3.86Gy. Due to the higher density of Dotarem compared to water, a new TP was developed and adjusted to account for the presence of the solution at the tumor, where a new energy layer was introduced. The integral dose to the Gd‐filled tumor was of 3.93±0.01Gy, and the $D_{90\%}$ was of 3.8Gy. The depth‐dose profile of the optimized TPs over the entire geometry, for the water and Dotarem target, can be seen in Figure S‐1 of the supporting material.

Upon proton irradiation, the primary protons interact with the medium they transverse, leading to the creation of secondary particles. The secondary photon spectra can be measured outside of the patient's body. When the tumor volume is filled with Gd, the spectral response diverges from the one of water, presenting lines characteristic of the Gd element,^18^ Figure S‐2 of the supporting material. The 43 keV, $k_{\alpha 1}$, is the most prominent Gd‐specific line in the secondary photon spectra recorded, being therefore the one further analyzed.

### 3.2 Response of Gd enables the tracking of the TP delivery

In PBS, due to the temporal spacing between the delivery of the different TP points, the spectral response of the system to the irradiation with each of the PBs can be individually evaluated. Therefore, the delivery of the TP can be followed by evaluating the Gd characteristic x‐ray signal resulting from the irradiation of the target by each PB in the TP.

**FIGURE 2:**
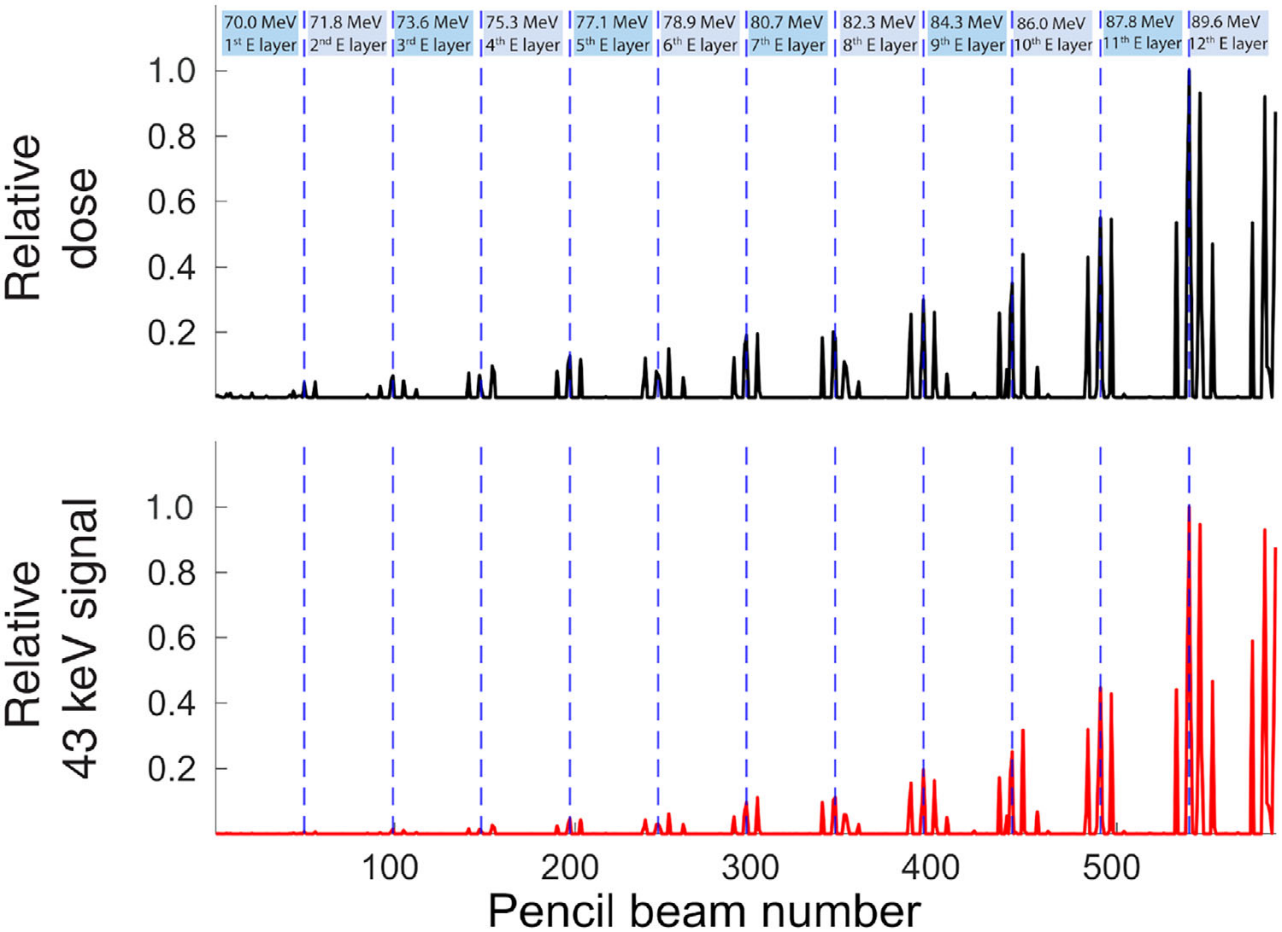
Relative dose delivered to the Gd‐filled tumor by each of the PBs in the TP (top) and the correspondent secondary 43 keV characteristic Gd x‐ray signal emitted (bottom), allowing TP tracking. Gd, Gadolinium; PBs, pencil beams; TP, treatment plan.

Both the dose delivered by each PB to the Gd‐filled tumor and the corresponding x‐ray emission upon irradiation were measured in the MC simulation. The TP points that most contribute to the dose coverage of the target were the ones of the high‐energy plane, 89.6 MeV, contributing to 42% of the total final dose at the target, Figure 2. Moreover, the PBs centered at the corners of the Gd volume, positioned at (−9,−9), (−9,9), (9,9) and (9,‐9) cm, exhibit the highest weight across the entire TP, and contribute approximately 74% of the total dose delivered to the target. When analyzing the x‐ray spectral response of the system individually for each scanning point, a similar behaviour is observed. Half of the total characteristic Gd signal, ca. 54%, is detected as a consequence of irradiation with the high energy layer of the TP, while 71% of the all spectral response recorded comes upon irradiation with the rays centered at the corners of the Gd cube, Figure 2. A good agreement is obtained between the dose contribution from each PB and the 43keV photons produced as a consequence of such irradiation.

### 3.3 Gd spectral response pinpoints deviations due to uncertainties

The target volume was then displaced over incremental steps in the transversal (*x*‐axis) as well as in depth (*z*‐axis) directions regarding the beam direction, in order to recreate organ motion, positioning errors, and geometry changes, which contribute to dose uncertainties.

The impact of displacements over the transversal plane is better visualized at a geometric level. Consequently, the PBs were grouped according to their origin position in order to facilitate the subsequent analysis.

**FIGURE 3:**
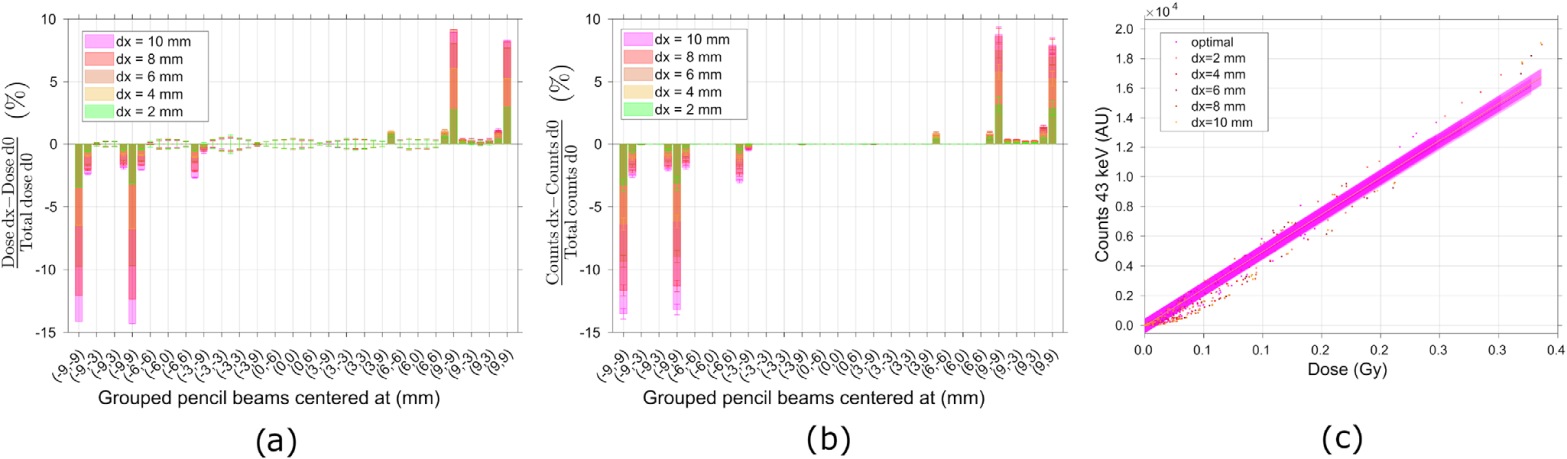
Deviations in (a) dose and (b) 43 keV Gd signal from the planned scenario due to transversal movements of the target. (c) Correlation between dose to the target given by each PB of the TP and the correspondent Gd 43 keV signal scored, for several transversal displacements of the tumor. Gd, Gadolinium; PBs, pencil beams; TP, treatment plan.

As the tumor volume is displaced in the transversal direction, the PBs in the negative corners centered at (−9,−9) cm and (−9,9) cm, deposit successively less dose in the tumor volume, since the target becomes increasingly out of their field, Figure 3a. Both PBs deposit less 14% of dose in the target when the tumor is displaced by 10 mm. An according behaviour is seen in the spectral response, where the 43keV signal of the (−9,−9) cm and (−9,9) cm centered PBs successively decreases for higher positive displacements, as such beamlets produce less interactions with Gd, Figure 3b. They produce less 13.2±0.4% of 43keV Gd signal recorded outside of the patient body, when it is displaced by 10 mm. Contrarily, the PBs centered at the positive corners of the Gd volume, increased their contribution to the overall deposition of dose in the target, Figure 3a. For a 10 mm displacement, all the PBs centered at (9,−9) and (9,9) contributed more 9% and 8%, respectively, of dose to the target. Such behaviour is followed by the detected characteristic x‐ray signal, which increases 9% and 8%, respectively, when the target is moved 10 mm, due to interactions of the same PBs. This relationship indicates a direct correlation between variations in the deposited dose at the tumor and the subsequent variations in the Gd characteristic signal detected for several transverse displacements.

A linear correlation was identified between the dose deposited by each PB and the produced characteristic Gd signal. Furthermore, the aforementioned relationship was maintained even when the target was displaced in the transverse plane, Figure 3c.

**FIGURE 4:**
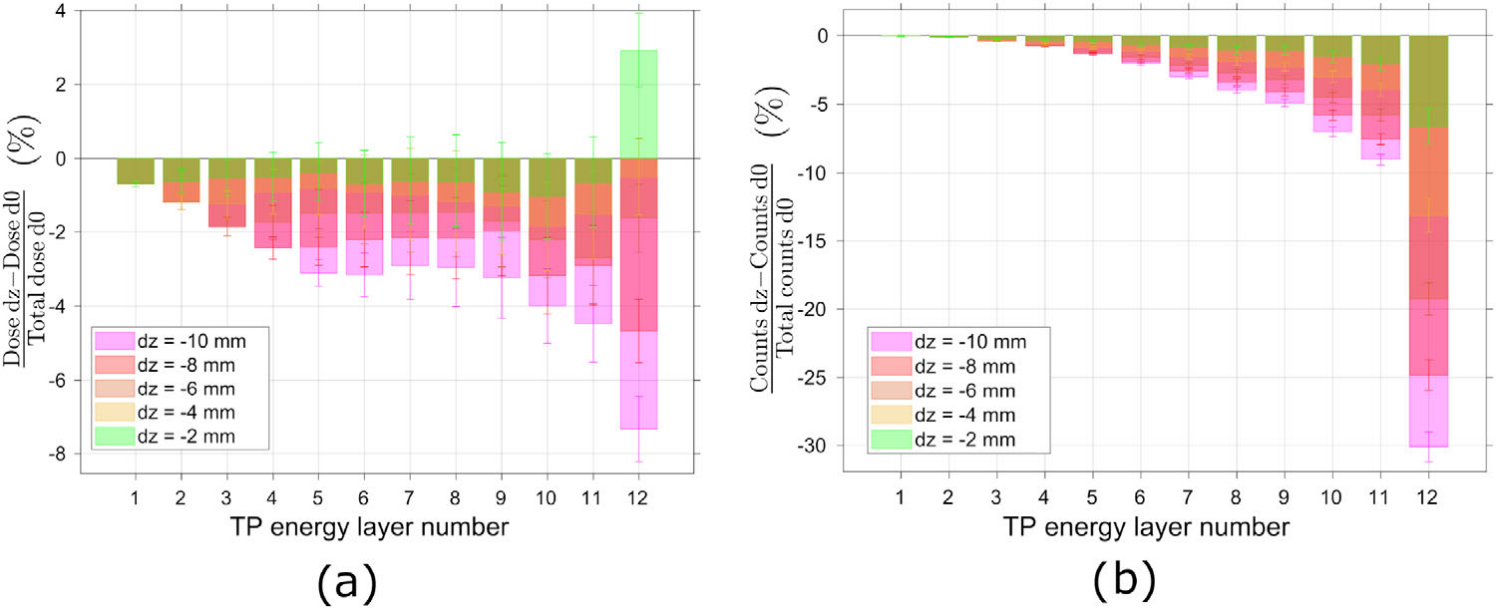
Deviations in (a) dose and (b) 43 keV Gd signal from the planned scenario due to downstream longitudinal movements of the target.

Contrarily to the evaluation of transversal displacements of the target, the impact of incremental steps in the longitudinal direction, *z*‐axis, can be more accurately assessed by grouping the PBs in energy planes. This approach reveals that all the energy planes deposited progressively less dose in the target when it moves successively downstream, Figure 4a. The higher‐energy layer's PBs, which have a higher overall weight in the TP, deposit less 3%, 5%, 7%, 11%, and 14% of dose in the target when it moves 2, 4, 6, 8, and 10 mm downstream, respectively. The Gd signal, also decreases successively for incremental displacements in the downstream direction, Figure 4b. The detected 43 keV signal decreases in 8%, 15%, 22%, 28%, and 34% for a 2, 4, 6, 8, and 10 mm downstream displacement, respectively.

**FIGURE 5:**
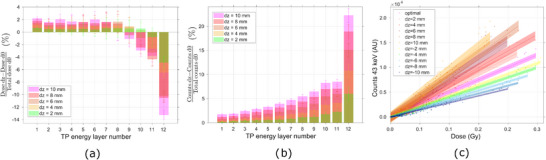
Deviations in (a) dose and (b) 43 keV Gd signal from the planned scenario due to upstream longitudinal movements of the target. (c) Correlation between dose to the target given by each PB of the TP and the correspondent Gd 43 keV signal scored, for several longitudinal displacements of the tumor. Gd, Gadolinium; PBs, pencil beams; TP, treatment plan.

On the other hand, when the target moves upstream, the lower energy TP points increase their contribution to the overall deposition of dose on the target, while the higher energy PBs deposit less dose on the target, Figure 5a. For the maximum displacement of 10 mm, there is more 13% in overall dose deposited by the first eight energy layers of the TP, where each energy layer is depositing individually up to 2% of dose in the target, and less 1%, 3%, 4%, and 13% of dose deposited in the target by the 9th, 10th, 11th, and 12th energy of the TP, respectively. Considering again the TP points of the higher energy layer, their contribution to the deposition of dose in the target decreases in 4.9%, 6.4%, 10.2%, 10.5%, and 13.2%, for a upstream displacement of 2, 4, 6, 8, and 10 mm, respectively. The detected Gd x‐ray signal, on the other hand, increases progressively for higher displacements of the target in the upstream direction, Figure 5b. For the full displacement of 10 mm all the energy layers produce more 43 keV x‐rays than in the optimal scenario. There is an incremental increase from 2% until 8% of the measured Gd signal, for the PBs of each of the first eleventh energy layers and an increase of 22% on the measured Gd‐signal arising from irradiation under the higher energy layer points. Contrarily to the behavior of deposited dose, when considering the response of the system under irradiation of only the last energy points of the TP, the detected Gd signal increases in 6.0%, 10.8%, 15.0%, 18.9%, and 22.2% for increasing displacements of the target of, respectively, 2, 4, 6, 8, and 10 mm in the direction of the beam nozzle.

**FIGURE 6:**
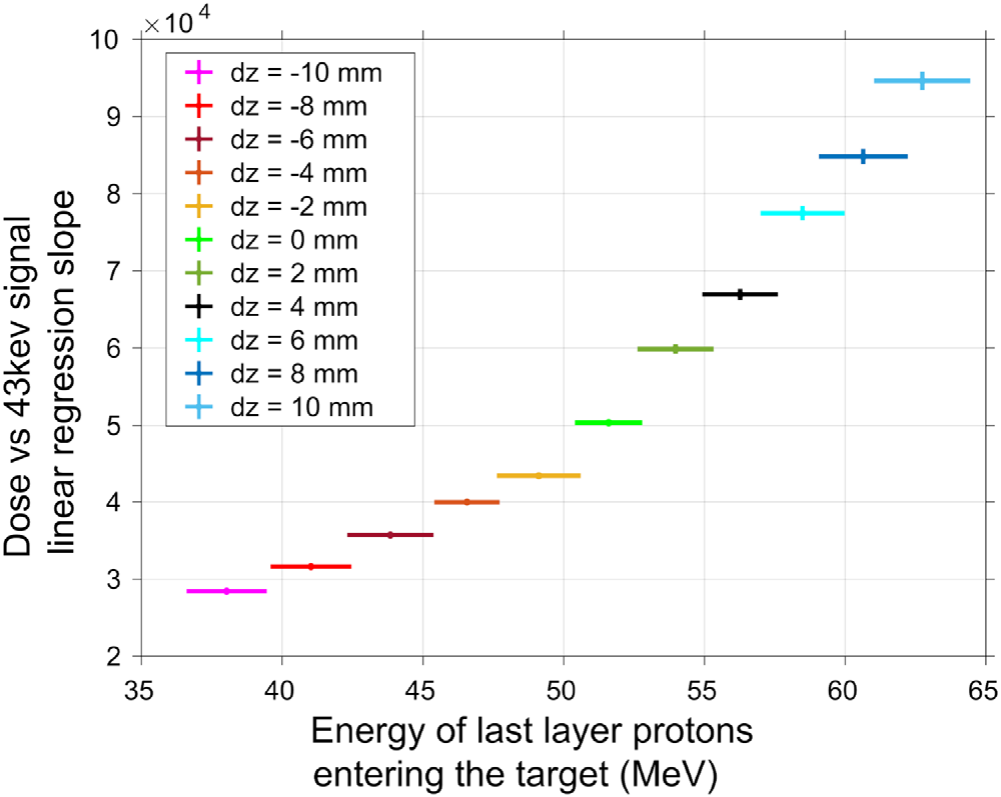
Correlation between the kinetic energy of the protons belonging to the last energy layer of the TP when arriving at the tumor volume and the slope of the data represented in Figure 5c. TP, treatment plan.

A linear correlation was once more observed between the dose deposited by each PB and the recorded Gd signal when the tumor was subjected to irradiation with the same TP while undergoing longitudinal movement. Although, contrarily to the behavior observed in transverse displacements, PBs with similar dose depositions at the target produce different x‐ray yields, depending on the longitudinal displacement they have undergone, Figure 5c. Consequently, the slope of the curves correlating the deposited dose with the spectral Gd response varies for each scenario. This slope can be correlated with the position of the target, and therefore with the kinetic energy of the protons arriving at the tumor volume. The slope of the correlation between the deposited dose by each PB and the corresponding Gd x‐ray signal is observed to increase with progressively downstream positioning of the target, which corresponds to an increase in the kinetic energy of the protons of the last energy layer of the TP arriving at the tumor volume, Figure 6.

The full TP can be monitored by assessing the cumulative response of the system, since in a PBS system the delivery of the full TP must match the cumulative contribution of all the PBs.

**FIGURE 7:**
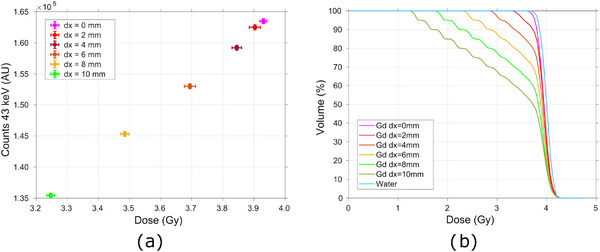
(a) Evaluation of the impact of transversal displacements of the target on the full TP delivery, by correlating the integral dose to the target given by the delivery of the full TP and the 43 keV Gd signal recorded; (b) Dose‐volume histogram for the Gd‐filled tumor irradiated with the full scanning TP with a prescribed dose of 4 Gy for the several transversal displacements of the target. Gd, Gadolinium; TP, treatment plan.

In a passive beam system, the delivery of the TP can only be evaluated as the result of the whole volume irradiation. Nevertheless, information about the fraction dose delivered could be obtained by analyzing the Gd spectroscopic response. In a PBS system this would correspond to the cumulative contribution of all the PBs. Therefore, the complete TP can also be monitored by assessing the cumulative spectroscopic response of the system. A linear relationship was observed between the integral dose to the target and the detected x‐ray Gd signal for transverse displacements of the target when the full treatment delivery is analyzed, Figure 7a.

Looking at the dose‐volume histograms (DVHs), one can see that the volume receiving 95% of the planned dose, decreases successively with tumor displacement, Figure 7b. In the optimal conditions 90% of the tumor volume is receiving 95% of the planned dose (3.8Gy). However, when the tumor moves 10mm in the transversal plane, only 46% of the whole target volume receives the same amount of dose. In fact, a displacement of only 2 mm already produces a 5.5% decrease in dose. It is important to note that no margins were applied when creating the optimized TP. These changes in the dose volume coverage and integral dose are followed by changes in the x‐ray spectra recorded, Figure 7a, which shows a decrease even for the smaller displacement considered of 2mm.

**FIGURE 8:**
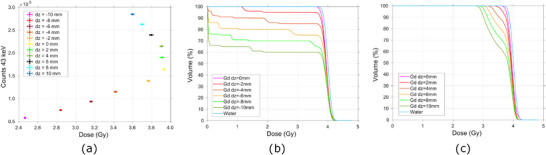
(a) Evaluation of the impact of longitudinal displacements of the target on the full TP delivery, by correlating the integral dose to the target given by the delivery of the full TP and the 43 keV Gd signal recorded; and Dose‐volume histogram for the Gd‐filled tumor irradiated with the full scanning TP with a prescribed dose of 4 Gy for the several (b) downstream longitudinal displacements of the target and (c) upstream longitudinal displacements of the target. Gd, Gadolinium; TP, treatment plan.

For longitudinal deviations of the target, a linear relationship between the integral deposited dose and the Gd spectral response is also observed for downstream movements. On the other hand, for upstream movements, the total spectral response of Gd increases for decreasing doses to the target. Two regions of response can be differentiated, Figure 8a.

As illustrated in Figure 8b, the DVHs demonstrate that for downstream displacements of the target, the volume receiving 95% of the prescribed dose decreases from the initial 91% to 56% in a −10mm displacement scenario. In this situation, the target is positioned successively beyond the range of the incident beam. Consequently, the dose received by 95% of the tumor volume decreases by 6% for a 2mm, by 85% for a 4mm shift and by 100% for the further shifts. The decrease in the integral dose to the target is followed by direct variations in the x‐ray spectra recorded, Figure 8a, which decrease 15%, 30%, 43%, 54%, and 65% for a 2, 4, 6, 8, and 10mm downstream shifts, respectively.

In the case of upstream displacements of the target, Figure 8c, the volume receiving 95% of the prescribed dose decreases from the initial 91% to 52% in a 10mm displacement scenario. Although, since the target moves to the build up region of the depth‐dose profile, for a 2 mm shift, the dose received by 95% of the its volume decreases by only 3%, and for the highest shift of 10 mm, it decreases by 21%. In this case, the decrease in the integral dose is followed by inverse variations in the x‐ray spectra recorded, Figure 8a, which increases 16%, 31%, 46%, 60%, and 73% for a 2, 4, 6, 8, and 10mm upstream shifts, respectively.

## 4 DISCUSSION

Uncertainties are a well known source of error in the delivery of proton TPs, compromising dose conformity to the tumor region and the safety of healthy tissues. Although, there is still no mechanism of accessing dose information in vivo and in real time during treatments. In this study we investigate a recent approach to track the dose delivered during the course of a treatment.

Due to the tumor's vascular permeability to macromolecules, a GBCA will be accumulated within the tumor tissue after its injection in the patient's body. The majority of the GBCAs are extracellular fluid agents that after intravenous injection, travel to the heart and then out to the systemic arteries, leaking into the extravascular extracellular space.^19^ These agents are rapidly eliminated from the body through the renal pathway. The range of the planned proton PBs and, consequently, the dose delivered to the tumor can be influenced by the presence of the GBCA, as it will alter the density of the tissue. However, the tumor concentration of Gd at the time of treatment will be dependent on the tumor biology, the physico‐chemical properties of the contrast agent and its washout time.^37^ Some studies have estimated an upper limit of Gd absorption within the tumor of 0.3 mg/g, and a clinical study has reported mean tumor uptakes between 0.012 and 0.17 mg/ml for a novel Gd‐based nanoparticle.^26^, ^38^, ^39^ Consequently, we predict that this effect will be negligible and not result in a need for re‐planning before treatment due to the administration of the Gd agent. Nevertheless, further research in this area would be beneficial.

The secondary photons that leave the patient's body can be detected in real time with an external out of the field detector, not influencing in any way the beam delivery process. The $k_{\alpha 1}$ characteristic x‐ray line, appearing in the low energy region of the Gd specific spectral signature is quite prominent in the proton irradiated Gd spectra, which hints at the suitability of tracking these x‐ray photons over the whole TP delivery as a surrogate for dose measurements.

Characteristic x‐ray photons can arise due to several processes, the most relevant of which are thermal neutron capture and PIXE. Studies are being carried out to understand such contributions and the underlying phenomena relevant to the studied technique. Characteristic x‐rays can arise as a product of the vacancy left after the ejection of an IC electron, releasing the energy of a compound nucleus, 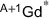, after thermal neutron capture. Alternatively, in the PIXE process, x‐rays are emitted due to the direct impact of a primary beam proton, which creates a vacancy in the electronic shell, leading to the characteristic x‐ray emission following atomic deexcitation.

In the case of PIXE, the number of ionizations produced in a given shell of a certain atomic species is proportional to the target concentration of that atomic species, number of particles having crossed the target, thickness of the sample and ionization cross section for a specific electronic shell.^40^ The cross section for the production of a characteristic x‐ray will then be given by the product of the ionization cross section and the fluorescence yield for that given shell.^40^ Based on this simple approximation of the phenomenon, the emitted characteristic x‐ray signal is proportional to the dose; depends on the target atomic number, concentration, and volume, and varies with energy, given that the ionization cross‐section depends on the energy of the incident particle.

On the other hand, Gd has a high thermal neutron capture cross section, mainly due to the presence of the 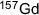 and 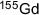 isotopes. In proton therapy, secondary neutrons are produced due to proton‐nuclear reactions in the materials along the proton path, both external and internal to the patient body. In an active system based facility the fluence of the externally produced neutrons is lower than in passive systems, due to the absence of components in the beam path, such as energy degraders, scatterers, and collimators.^41^, ^42^ In the simulation carried out for this study, only the neutrons produced inside the patient body (approximated to the water phantom) were considered and tracked down. Therefore, a fraction of neutron capture events that would occur in a clinical setting were not accounted for in this analysis. The secondary neutron production is dependent on the proton dose delivered to the patient, the energy of the incident protons, and the patient body geometry. The thermal neutron capture yield will be dependent on the number of Gd nuclei per unit volume, the volume transversed by the neutrons, the capture cross section, and the amount of thermal neutrons produced. Additionally, the total yield of IC electrons per neutron capture in Gd is 0.5991.^43^ Subsequent to IC, Auger electrons and characteristic x‐rays are emitted following the relaxation of the Gd atom to the ground state.

The results obtained demonstrated the feasibility of monitoring the delivery of the TP. Information regarding the dose deposited by each PB could be accessed through spectral analysis, Figures 2 and 3c. In a PBS based facility, the dose distribution of each treatment field is subsequently built up by a multitude of PBs of different positions and energies. Therefore, if the dose deposited by each PB is assessed during treatment, the TP can be adapted^7^ by implementing changes to the TP, either within or between fractions, compensating for deviations from the optimal scenario.

Moreover, alterations to the overall patient geometry can also be distinguished by analyzing the variations in the spectral response of the system. For horizontal and vertical movements of the target ‐ in the transversal plane ‐ the decrease/increase in dose delivered by one PB always translated to a proportional decrease/increase, respectively, in the Gd x‐ray signal recorded, Figure 3a and 3b. If the volume moves away from the designated treatment region (as into the positive *x*‐direction) it will subsequently be positioned further outside the field for the PBs centred at the ‐x positions, which will contribute successively less dose to the target, reducing the tumor dose volume coverage, Figure 7b. In the case of the PIXE process, the Gd characteristic signal is a function of the concentration and volume of the Gd target and of the quantity of particles crossing it. Therefore, the reduction in volume coverage will be reflected in a decline of the Gd x‐ray signal, proportional to the variation in dose. Considering the portion of the signal of interest that arises subsequent to neutron capture events, a direct analysis is not so straightforward. Thermal neutrons are mainly generated internally in the vicinity of the target area and emitted isotropically. Their fluence diminishes with increasing distance from the target in all directions, resulting in a nonlocalized deposition of energy that extends beyond the proton beam location and into surrounding tissues.^25^, ^44^ Therefore, one can predict that the 43 keV signal resulting from neutron capture will decrease for high displacements of the target. However, for minimal shifts in target position, the difference in thermal neutron fluence may be insufficient to be expressed as a change in the Gd x‐ray spectra.

In the event of a longitudinal displacement of the target, variables such as the energy of the protons upon reaching the Gd‐filled tumor and the number of interacting particles will have a higher impact on the Gd system response. As the primary protons interact with the medium, they slow down mainly due to countless interactions with atomic electrons, although they can also be deflected due to interactions with atomic nuclei in multiple Coulomb scattering or undergo nonelastic nuclear collisions.^4^ As so, the proton kinetic energy as well as its fluence will be depth dependent. When the target moves away from the beam nozzle, a part of its volume is placed outside of the protons' range. For example, for a −10 mm displacement, 1/3 of the Gd‐filled‐tumor length is out of the protons' range, Figure 8B. The tumor is therefore partially placed in the distal region of the SOBP, where the deposition of dose is minimum. Furthermore, the mean kinetic energy of the incident protons will also suffer a displacement to lower energies as depth increases, which impacts the ionization cross section of the Gd atoms. According to PIXE, both these factors contribute to a reduction in the emitted Gd x‐rays. Additionally, the neutron fluence increases with depth up to two‐thirds of the Bragg peak, at which point it begins to decline due to cessation of secondary particle creation. The high‐energy neutrons are forward‐directed relative to the primary protons, while the low‐energy neutrons are more isotropic.^45^ Therefore, the secondary neutron flux density is concentrated in the forward direction with smaller statistics outside of the beam direction.^46^ As so, the relative contribution of the thermal neutron capture phenomena for the recorded signal is expected to increase for downstream displacements.

On the other hand, when the target moves in the direction of the beam nozzle, it will be placed in a shallower region of the SOBP, where the dose deposited is lower due to the lower stopping power of the incident protons. When the tumor moves in such direction, its whole volume is still within the range of the primary protons, and, therefore, there will be no variations in the percentage of tumor volume crossed by the PBs, as in the previous cases. However, the mean kinetic energy of the incident protons will increase for successive upstream displacements, as will the fluence of primary protons arriving at the tumor. Literature data on Gd k‐shell ionization cross‐sections by incident protons can be found for energy ranges up to 15 MeV.^47^, ^48^ In these energy intervals, which can be seen at the end of the clinical proton ranges, the data indicate that the cross section from PIXE increases with increasing kinetic energy of the incident particles. Therefore, the intensity of the recorded Gd x‐ray signal is expected to increase, even if the deposited dose to the target is lower. This method could be combined with prompt gamma spectroscopy methods, for example, to retrieve the energy of the primary protons and directly obtain the dose at the target, given by the Gd characteristic x‐ray signal.

As in movements of the target, variations in the path of the beam, like an air cavity, filling of cavities with anatomical fluid, a metal implant, or a bone structure, will also produce variations in the kinetic energy and fluence of the arriving protons at the target, and consequently on the range of the PBs. Therefore, one can anticipate observing similar variations in the measured Gd signal in such cases, which suggests that daily anatomic variations can also be tracked.

Moreover, in light of both PIXE and thermal neutron capture phenomena, the spectral response of the system will also be influenced by the concentration of Gd present, being expected a decrease in the characteristic Gd x‐ray signal with a decrease in the GBCA concentration. The determination of the concentration of Gd at the tumor site at the time of treatment can be important to parameterize the system response. A quantitative MRI analysis that correlates T1 times and Gd concentration,^49^, ^50^, ^51^ or prompt gamma spectroscopy, could possibly be used to retrieve this information prior to treatment.

In abdominal patients, inter‐ and intra‐fraction uncertainties from setup positioning, breathing, and gastrointestinal content variations significantly impact treatment. For pancreatic patients, the proximity of the pancreas to radiosensitive organs at risk (OARs), such as the stomach and duodenum, complicates high‐dose radiation delivery to the tumor without increasing OAR exposure.^52^ In prostate patients, anatomical variations like bladder filling and rectal motion cause prostate movement, introducing dose uncertainties due to tissue density variations along the beam path. Femoral bones can affect dose distribution when using lateral beams.^6^ Precise control of TP delivery is crucial for these sites. However, the higher thickness of the abdominal region may attenuate Gd secondary x‐rays and limit the method's applicability. The relevant energy peak at 43 keV has a half‐value layer (HVL) of approximately 2.6cm in water,^53^ suggesting external detection might be hampered by body absorption, necessitating an additional attenuation correction step. A patient‐specific evaluation should consider tumor depth and surrounding tissue thickness.

In lung patients, the most significant proton path length alterations are due to tumor movements within the lung tissue, influenced by respiration and tumor shrinkage. Lung tissue's lower density causes greater dose shifts compared to more water‐equivalent tissues. The lung itself is a dose‐limiting OAR,^54^ and organs like the spinal cord or esophagus can show considerable DVH fluctuations due to setup errors.^55^ Techniques like 4D planning and breath gating help mitigate these uncertainties; however, monitoring the TP delivery remains crucial.^54^ The lower density of lung tissue, with varying aeration degrees, leads to a reduced attenuation of Gd characteristic x‐rays, since their HVL lays between 2.45 and 2.9cm in lung tissue and 2314 and 2765cm in air,^53^ enhancing detection efficiency and signal‐to‐noise ratio. Consequently, superior performance of this method for lung patients is expected.

Head and neck patients face setup uncertainties and anatomical changes, such as nasal cavity filling and patient weight loss during treatment, complicating accurate TP delivery due to the region's complex and heterogeneous anatomy.^56^, ^57^ Close monitoring of TP delivery is essential. The reduced tissue thickness surrounding the tumor enhances the probability of x‐ray detection, although surrounding bone may interfere, as the 43 keV x‐ray HVL in bone is between 0.54 and 0.85cm.^53^

Finally, techniques as stereotactic body proton therapy^58^, ^59^ or the novel researched area of FLASH,^18^, ^60^ where higher doses are delivered in fewer fractions or at a higher dose rate, could facilitate the technique. Given the high doses employed, a sufficient detectable signal may remain even after tissue attenuation. In the case of FLASH, this technique would also address the gap brought by the high dose rates employed, since the standard dosimetry instruments like ionization chambers, fail at such rates due to saturation.

## 5 CONCLUSION

This study examines the potential of the use of Gd as a surrogate agent for in vivo dosimetry during scanning proton therapy. Gd is introduced into the tumor volume through the injection of a GBCA in the patient prior to treatment, which accumulates preferentially in the tumor volume due to its leaky vasculature. Therefore, the effect of the planned TP can be assessed directly within the tumor region, eliminating the necessity for extrapolating the dose at the tumor site from a value at an entrance tissue or cavity in the vicinity, enhancing the precision of this assessment.

In summary, the delivery of a TP was successfully tracked by following the secondary 43 keV x‐ray signal characteristic of Gd. Inter‐fraction variations, such as positioning errors, tumor shrinkage, or different anatomic fillings, could be identified through variations in the full Gd signal recorded. Intra‐fraction movements, such as breathing or other geometrical deviations, could be tracked through the spectroscopic analysis of the effect of each individual PB. Therefore, a qualitative analysis is possible comparing the Gd spectral response during or after the course of a treatment with the expected response based on the TP and taking into account a specific patient geometry. This opens the possibility to adapt the delivered TP intra‐ and inter‐fraction, reducing uncertainty margins, increasing the tumor control probability and reducing the normal tissue complication probability.

However, the proposed dosimetry method is a multi‐variable approach, and a direct quantitative analysis from the Gd spectroscopic signal recorded calls for a precise parametrization of the variables contributing for the signal of interest. One of such variables is the concentration of GBCA in the tumor site during treatment. Patient anatomy will also affect the measured signal, as the thickness and density of the tissues between the Gd‐filled tumor and the detector determine the attenuation of the characteristic x‐rays. An attenuation correction may have to be done in order to achieve a quantitative value for the deposited dose at the tumor. As so, absolute measurements of dose, which are the ultimate goal of the investigated technique, require further research and a detailed knowledge of the factors that interfere with the recorded spectroscopic signal.

## CONFLICT OF INTEREST STATEMENT

The authors declare no conflicts of interest.

## Supporting information

Supporting Information
