# Supplementary material for: In vivo dosimetry for proton therapy: A Monte Carlo study of the Gadolinium spectral response throughout the course of treatment
Source: Med Phys. 2025 Jan 21;52(4):2412–24. doi: 10.1002/mp.17625 (PMC11972047; doi:10.1002/mp.17625)
Supplement: Supplementary file 1 — Supporting Information [file MP-52-2412-s001.pdf]

Supporting material

In vivo dosimetry for proton therapy: a Monte Carlo study of the Gadolinium spectral response throughout the course of treatment

M. Brás, H. Freitas, P. Gonçalves, J. Seco

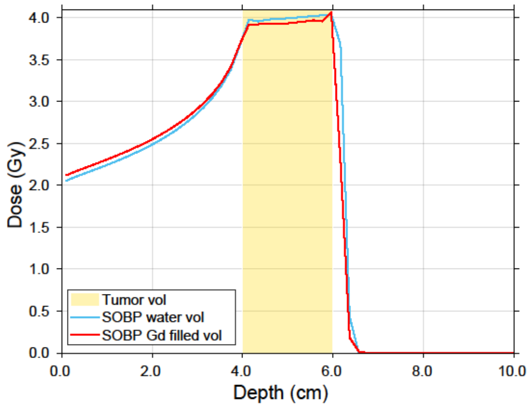

Figure S1: Dose-depth profile of the optimized TP over the considered geometry with a water (blue line) and dotarem (red line) inserted target.

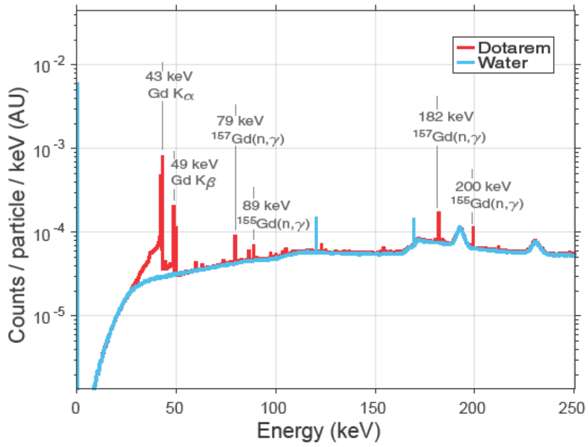

Figure S2: Spectra of the secondary photons emitted upon proton irradiation of a Gd (red curve), or water (blue curve) volume inserted in a water tank, in the low energy range. $k_{\alpha 1}$, $k_{\alpha 2}$ and k_{β} lines can be distinguished at energies 43.0 keV, 42.3 keV, and 48.7 keV, respectively, as well as a series of prompt gamma lines at 79 keV, 89 keV, 182 keV and 200 keV.
